# Individual and Herd-Level Seroprevalence in Association with Potential Risk Factors of Japanese Encephalitis in Pigs Collected from Urban, Periurban, and Rural Areas of Bali, Indonesia

**DOI:** 10.1155/2023/9682657

**Published:** 2023-02-15

**Authors:** I. Made Kardena, Anak Agung Ayu Mirah Adi, I. Nyoman Mantik Astawa, Mark O'Dea, Ian Robertson, Shafi Sahibzada, Mieghan Bruce

**Affiliations:** ^1^Department of Pathobiology, Faculty of Veterinary Medicine, Udayana University, Jalan PB Sudirman, Denpasar 80234, Bali, Indonesia; ^2^School of Veterinary Medicine and Centre for Biosecurity and One Health, Harry Butler Institute, Murdoch University, Perth, 6150, Western Australia, Australia; ^3^Department of Primary Industries and Regional Development (DPIRD), Diagnostics and Laboratory Services, Sustainability and Biosecurity, South Perth 6150, Western Australia, Australia

## Abstract

A study to assess the seroprevalence antibodies against JEV in pigs in Denpasar, Badung, and Karangasem as the representatives of urban, periurban, and rural areas in the province of Bali was conducted. Sampled pigs' blood was collected and their sera were tested for antibody detection using commercial IgG ELISA. A standard questionnaire was used to interview the pig owners or farmers to identify the determinants associated with the seropositivity of the antibodies. Overall, 96.6% (95% CI: 94.5–98.1) of 443 pig sera in individual animal-level seroprevalence were seropositive to the ELISA. Karangasem had the highest test prevalence at 97.3% (95% CI: 93.1–99.2) while Badung had a slightly lower prevalence at 96.6% (95% CI: 92.2–98.9), and Denpasar had the lowest prevalence at 96% (95% CI: 91.5–98.5) (*p*=0.84). In herd-level seroprevalence, all sampled herds contained one or more seropositive pigs (overall herd-level seroprevalence 100% [95% CI: 97.7–100]). No animal-level factors were significantly associated with seropositivity (all *p* values >0.05). For the herd-level risk factors relating to pig management and husbandry practices adopted, no analysis model could be generated, as all the sampled herds were seropositive. More than 90% seroprevalence detected in this study indicates high natural JEV infection occurred in pigs, which highlights the high public health risk of the infection in the areas.

## 1. Introduction

Japanese encephalitis (JE) is a viral zoonotic arthropod-borne disease caused by Japanese encephalitis virus (JEV) that belongs to *Flaviviridae* family [1, 2] along with other important arboviruses including dengue and West Nile viruses [3]. Although infection in humans generally results in minor clinical symptoms [4], an acute encephalitis syndrome (AES) can develop in less than 1% of the total infected people [5]. AES is characterized by an acute onset of fever and change of mental status, with a case fatality rate of 30%, and up to 50% develop long-term neurological disturbance [6, 7]. The JEV transmission in humans occurs through mosquito bites of the *Culex* genus, primarily *Culex tritaeniorhynchus* [8], by feeding on viraemia JEV infected pigs [9].

In general, JE cases in pigs in Southeast Asia occur in rural areas, where paddy fields and pig farms are widely available [1, 10]. However, there is growing evidence indicating that the cases are also found in urban and peri-urban areas. Evidence of JE has been reported from several cities and their vicinities in Southeast Asian countries including Can Tho, Ho Chi Minh, Hai Phong, Nha Trang, and Hanoi in Vietnam [11] and Phnom Penh in Cambodia [12]. However, there seem to be few studies that have investigated the disease frequency in animals in other rural, periurban, and urban areas [13], including in Indonesia [14].

The occurrence of the infection in pigs is influenced by many factors, including management and husbandry practices [2]. Investigations on the antibodies in pigs and their potential risk factor identification have been performed in several countries. Studies of the seropositivity antibodies against JEV that were associated with the animal-level and herd- or farm-level risk factors have been conducted in Malaysia [15] and Nepal [16]. In the USA, high demand in swine production, vector competence found in the pig farms, and the capacity and frequency of swine waste lagoon flooding as the vector mosquito oviposition site were the potential factors that associated with the emergence of the virus in pigs [17]. However, studies on antibody detection and risk factor identification in pigs in Indonesia, including Bali, are still very limited. In this investigation, a study was undertaken to determine the seroprevalence of antibodies against JEV in pigs from the regions of Denpasar, Badung, and Karangasem in Bali representing urban, periurban, and rural areas. Data were also collected on management and husbandry practices adopted to evaluate putative risk factors for the seropositivity of individual animals and herds containing one or more seropositive pigs.

## 2. Materials and Methods

### 2.1. Serological Survey of Pigs and Questionnaire Survey of Pig Farmers

#### 2.1.1. Study Area

A cross-sectional study was conducted from January to April 2020 in three areas of Bali, Indonesia: Denpasar city, Badung regency, and Karangasem regency. These three locations were purposefully selected because of a documented high incidence of AES cases in humans from 2016 to 2018 (Dinas Kesehatan Provinsi Bali, 2019). To determine the number of pigs to sample, the online software Epitools was used (https://epitools.ausvet.com.au/). Using an estimated prevalence of 0.32 [18], and desired of 0.05, a power of 0.8, and a confidence level of 95%, the total minimal sample pigs required was 335 for the three areas.

**Figure 1:**
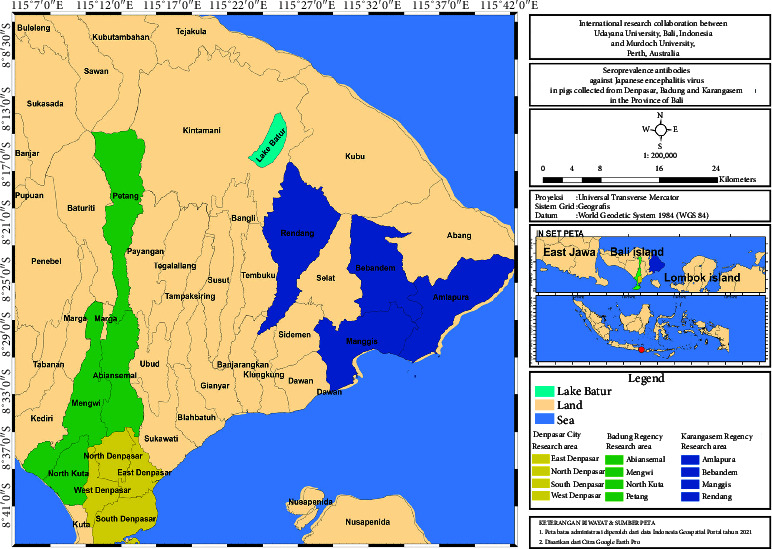
Map of selected sampling locations to estimate the seroprevalence of JEV in pigs from Denpasar city, Badung, and Karangasem regencies in the province of Bali, Indonesia.

Twelve subdistricts (four from each district) were selected: four of the six subdistricts from Badung (Abiansemal, North Kuta, Mengwi, and Petang), and four of eight subdistricts in Karangasem (Amlapura, Bebandem, Manggis, and Rendang) were randomly selected whilst all four subdistricts in Denpasar (West Denpasar, South Denpasar, East Denpasar, and North Denpasar) were included (Figure 1).

#### 2.1.2. Survey of Pig Farmers

A list of household pig farmers or owners from 2019 was obtained from the Pusat Kesehatan Hewan (Animal Health Posts) of the Dinas Pertanian (Department of Agriculture) for each of the three districts. Individual farmers/piggery owners from each of the twelve subdistricts were selected using simple random sampling.

Most farmers adopted a traditional rearing system; however, some used a semiintensive rearing system. In a traditional rearing system, pigs are allowed to roam freely or are sometimes tethered. If pigs were kept in a pen, the pen walls tended to be made of wood, bamboo, or brick with less than 10 pigs in the pen. In a semiintensive system, all pigs were confined with the pen walls and floor made of concrete, the total number of pigs kept was greater than 10, and the pigs' drinking water was sourced from the local municipality reticulated water system [19].

#### 2.1.3. Interview Using a Questionnaire

The questionnaire was divided into two sections and included 36 questions (Supplementary file available here). The first section focused on questions about the sampled pigs including their age, sex, breed, and vaccination status. The second section included 32 questions focused on the pig-farmers management and husbandry practices including herd size, if pigs were confined or not, why pigs were reared, the proximity of pigs to the farmer's house, frequency and method of cleaning pens and feed/drink containers, and frequency of feeding. The questionnaire interview was administered in Bahasa Indonesia, and the answers were subsequently translated into English for analysis. The interview was conducted on the same day as when the pigs were sampled.

#### 2.1.4. Ethics Approval and Informed Consent

Before collecting blood samples from pigs, the farmers/owners were informed of the purpose of the survey, why it was being undertaken and what the project hoped to achieve. Participants were advised that they could withdraw from the study at any point, their involvement was voluntary, and they would not be specifically identified in any analyses or reports. Informed consent to be involved in the project was obtained orally. On receiving this consent, pigs were sampled, and then, the farmers or owners were interviewed using the standardized questionnaire to determine basic husbandry and management practices implemented in their piggeries.

This research was approved by the Animal Ethics Committee of Murdoch University, Australia (R3207/19), and the Ethics Commission for the Use of Animals in Research and Education, Faculty of Veterinary Medicine, Udayana University, Bali, Indonesia (14/UN14.2.9/PD/2020). The research protocol was also approved by the Human Ethics Committee, Faculty of Medicine, Udayana University (233/UN.14.2.2.VII.14/2020).

#### 2.1.5. Blood Collection

The survey was conducted between January and April 2020. Two to five pigs from each herd were provided by the farmers, and then, their blood was collected.

Blood was collected from the jugular vein or anterior vena cava using a 3 or 5 mL syringe with a 23 G × 1.25-inch needle or a 22 G × 1.5-inch needle, respectively. Approximately, 2-3 mL of blood was collected from each sampled animal. The samples were allowed to clot at room temperature and then centrifuged at 4000 rpm for 10 minutes. The serum was then decanted and stored at −20°C for up to three months. A day prior to test the sera for JEV antibodies the samples were transferred from the freezer to a 4°C refrigerator to thaw.

### 2.2. Diagnostics and Analysis

#### 2.2.1. Diagnostic Procedure of Enzyme-Linked Immunosorbent Assay (ELISA)

A commercial IgG ELISA kit (E-AD-E002, Porcine Japanese Encephalitis Virus Antibodies ELISA kit, Elabscience, China) and an ELx 800-Biokit™, BioTek®, USA, and ELISA reader was used to detect antibodies against JEV in the pig sera. The ELISA has a sensitivity (Se) of 98.3% and specificity (Sp) of 98.2% (personal communication with Elabscience). The test was performed as per the manufacturer's instructions (https://www.elabscience.com/Manual/elisa_kits/E-AD-E002.pdf), and the optical density (OD) was read at 450 nm.

#### 2.2.2. Data Analysis

Animal- and herd-level test seroprevalence and real seroprevalence and their 95% confidence intervals (95% CIs) [20] were calculated. A herd was classed as positive if one or more of the pigs sampled in that herd tested positive. Differences in seroprevalence between the three regions sampled were analyzed with a Chi-square test for independence (Statistix 10, Analytical Software).

The influence of animal-level factors (age, sex, breed, and vaccination status) and herd-level factors (rearing system, purpose of keeping pigs, number of pigs per pen and herd, frequency to feed the pigs, frequency to clean the pigpen, methods used in cleaning the pigpens, water sediment found on pigpen floor, drainage around the pig herd, close proximity pig herd to the farmers' houses and rice paddy fields, and the presence of mosquitoes in the pigpens) on animal-level seroprevalence and herd-level seropositivity, respectively, were determined using data from the questionnaire. Initially, factors were assessed using a Chi square test or Fisher's exact test using OpenEpi (https://www.openepi.com/). Factors that had a *p* value ≤0.25 were included in a logistic regression model (IBM SPSS ® 23). The final model was built using backward elimination, with factors with a *p* value <0.05 remaining in the model. The fit of the data was assessed by evaluating the Hosmer–Lemeshow statistic and interaction between the reduced subset of variables assessed.

## 3. Results

A total of 457 pig serum samples from 162 pig herds were collected and tested for seropositivity to JEV. Fourteen of these samples (two from Denpasar, four from Karangasem and eight from Badung) were excluded from the study as they generated indeterminate results on the ELISA. Of the 443 suitable sera, 150 sera came from 55 herds from the four subdistricts of Denpasar city, 147 came from 50 herds from Badung regency, and 146 came from 57 herds from Karangasem.

Overall, 96.6% (95% CI: 94.5–98.1) of suitable samples were seropositive to the ELISA. The highest animal-level test seropositivity was observed in Karangasem (97.3%, 95% CI: 93.1–99.2) compared to 96.6% in Badung (95% CI: 92.2–98.9) and Denpasar (test seropositivity of 96%, 95% CI: 91.5–98.5) (Table 1). These animal-level seroprevalences were not significantly different (*p*=0.84).

All sampled herds (18 traditional and 144 semiintensive pig rearing management systems) contained one or more seropositive pigs (overall herd-level test seroprevalence is 100% [95% CI: 97.7–100]).

As there was no significant difference in the animal-level test seroprevalence between the three study areas, the animal-level model could be generated as combined data to test for the influence of animal-level factors on seroprevalence (Table 2). No animal-level factors were significantly associated with seropositivity (all *p* values >0.05).

As the herd-level seropositivity was 100%, herd-level risk factors could not be determined.

## 4. Discussion

The test prevalence of JEV antibodies observed in this study was very high at 96.6% (95% CI: 94.48–98.09). This result is similar to other related studies conducted in other provinces in Indonesia. The seroprevalence antibodies percentage against JEV in pigs in Riau province was found to be 94% (*n* = 190), while the same percentage was also observed in pigs (more than 6 months of age) (*n* = 69) in North Sumatra province, Indonesia [21]. Similarly, 99% (*n* = 73, 95% CI: 96%–100%) seroprevalence of the antibodies was observed in Vietnam [22]. Moreover, the same proportion of seroprevalence in pigs (96.6%, *n* = 29) was also found in a study performed in Cambodia [12]. This high seroprevalence detected indicates a high rate of natural JEV infection occurring in pigs, which is of concern given their role as amplifying hosts for the virus.

The seroprevalence has increased in pigs in Bali over the past two decades from 32% in 2006 [18] to 49% in 2008 [23] and from 60% in 2015 [24] to 96.6% in this study. This suggests that the JEV circulation, transmission, and infection in pigs are getting more intensive in the area.

In this study, the pigs less than six months of age in all three districts were found to have a high test prevalence at 95.8% (95% CI: 92.9–97.7). This suggests that JEV infection likely occurred in piglets in their first six months of life. In the JEV endemic areas, especially in Southeast Asian countries, a high proportion of antibodies against JEV in the young pigs before reaching their sexual maturity is commonly found [10, 12].

In similar, high test seroprevalence of the antibody was also detected at more than six months of age in pigs collected (98.5%, 95% CI: 94.7–99.8). However, this antibody detected might likely be triggered by the infection that occurred at an early age and persists for several years [25]. Moreover, pigs tend to have repeated natural infections resulting in continued elevated antibody levels in JE endemic area [26].

The high proportion of the antibodies detected in young and adults pigs in this study suggests a high intensity of JEV circulation that not only occurs in the rural area of Karangasem, but also in the peri-urban area of Badung regency and the urban area of Denpasar city in Bali. Unlike some studies which have reported that JE predominantly occurs in rural areas [27–29], in Bali the seroprevalence in pigs from urban areas was just as high as that in pigs from rural areas. The findings of high seropositivity in pigs from urban areas are consistent with those in other south-east Asian and subcontinent countries including Vietnam [11, 22, 30], Cambodia [10, 12, 31], and India [26, 32, 33]. The high seroprevalence of JEV across the districts indicating the frequent transmission of JEV highlights the potential for JE transmission to humans in urban areas where little agricultural activities are undertaken, with few pig farms or rice paddy fields, demonstrating the need to consider broader socioecological factors contributing to transmission [34].

Although the three areas included in this study of Denpasar, Badung, and Karangasem are administratively and geographically different, their socioecological factors are similar, which likely contributes to the similar high seroprevalence in the sampled pigs. Denpasar city is located in the southern part of Bali and borders Badung regency. Karangasem regency is located approximately 80 km northeast of Denpasar. Residents tend to keep pigs for their local ceremonies. Even in Denpasar city Balinese rear pigs, although the size of the pig population is much lower than that in Badung and Karangasem regencies. In 2018, the pig density in Denpasar was recorded to be 112.5 pigs per square km, while in Badung and Karangasem regencies the densities were 168 and 170 pigs per square km, respectively [35].

Rice paddy fields are common in Badung and Karangasem, but rare in Denpasar. However, the presence of stagnant water, poor drainage, and open sewage sites in Denpasar offer ideal locations for the mosquitoes to breed in urban areas, increasing the likely occurrence of mosquito-borne diseases, including JE [36]. Several studies have identified the presence of the necessary mosquito vectors for JEV in Bali [14, 37–39]; however, further studies are required on mosquitoes that feed on pigs and hence are potentially involved in the transmission of JEV to humans.

Given the high density ratio of 4 : 1 of humans vs. pigs in Bali [23, 40], as well as the close proximity of human dwellings to pig pens [24, 38, 41], it is not surprising that JEV is a common and important disease in humans on the island. Pig population density seems to be another important factor [42] that favours JEV transmission between pigs as well. However, the density factor in each of the study areas was not evaluated in this study and it could be one of the future directions that need to be investigated for similar studies.

No determinants or risk factors at the animal-or herd-level were associated with an increased risk of seropositivity. The extremely high animal- and herd-level seroprevalence was the cause of this. In contrast, studies conducted in Malaysia [15] and Vietnam [43] found that the seroprevalence in pigs increased with age, most likely associated with an increased opportunity to be bitten by an infected mosquito over time.

It is possible that the seroprevalence was overestimated in this study [44, 45] as the antibodies detected were not confirmed with the recognized gold standard neutralizing test due to a lack of a biosafety level 3 laboratory. Some false positive results might be occurred due to cross-reaction with any of the JEV serogroup flaviviruses, such as dengue [46] and Zika [47] viruses which are also detected in Bali. However, the use of the recombinant JEV for precoating of the ELISA plates may have reduced the occurrence of cross-reactions [48].

## 5. Conclusion

More than 90% seroprevalence of JEV in pigs detected in this study indicates a high rate of natural JEV infection occurring in pigs that collected from the urban area of Denpasar city, periurban area of Badung regency, and rural area of Karangasem regency in the Province of Bali. Pigs seem to have a major role as a JEV source and for transmission of the disease, which consequently highlights the high public health risk of the infection in the areas.

## Figures and Tables

**Table 1:** Animal-level test seroprevalence and real prevalence of JEV in pig sera collected from the regencies/city of Denpasar, Badung, and Karangasem in the province of Bali.

Regencies/city	Subdistricts	Number of positive samples	Total number of samples tested	Test prevalence (95% CI)	Real prevalence^*∗*^ (95% CI)
Denpasar		144	150	96% (91.5–98.5)	97.6% (97.6–98.5)
	South Denpasar	40	40	100.0% (91.2–100)	100% (95.4–100)
	East Denpasar	37	38	97.4% (86.2–100)	99% (90.8–100)
	West Denpasar	41	45	91.1% (78.8–100)	92.6% (92.1–97.5)
	North Denpasar	26	27	96.3% (81–100)	97.9% (87.2–100)

Badung		142	147	96.6% (92.2–98.9)	98.2% (97.5–98.9)
	Abiansemal	18	23	78.3% (56.3–92.5)	79.2% (85.2–92.5)
	North Kuta	62	62	100% (94.2–100)	100% (94.2–100)
	Mengwi	33	33	100% (89.4–100)	100% (89.4–100)
	Petang	29	29	100% (88.1–100)	100% (88.1–100)

Karangasem		142	146	97.3% (93.1–99.2)	98.9% (97.5–99.2)
	Amlapura	36	37	97.3% (85.8–100)	99% (90.5–100)
	Bebandem	43	43	100.0% (91.8–100)	100% (91.8–100)
	Manggis	37	38	97.4% (86.2–100)	99% (90.8–100)
	Rendang	26	28	92.9% (76.5–99.1)	94.4% (87.7–99.1)

^*∗*^Calculated by adjusting test seroprevalence using 98.3% sensitivity and 98.2% specificity of the assay.

**Table 2:** Potential association of individual animal-level risk factors of age, sex, breed, and vaccination status with test seroprevalence to JEV.

Risk factors	Number samples	Seropositive (%)	Risk ratios (95% CI)	*p* values^#^
Age				
≥6 months	134	98.5	1.028 (0.99–1.00)	0.12^*∗*^
<6 months	309	95.8		
Sex				
Male	128	97	1.004 (0.97–1.00)	0.45
Female	315	96.5		
Breed^*∗∗*^				
Landrace	300	97.7		
Others	143	96.7	1.035 (0.99–1.00)	0.14^*∗*^
Vaccination status^##^				
Unvaccinated	75	97.3		
Vaccinated	368	96.7	1.006 (0.96–1.00)	0.45

^*∗*^RR > 1 and *p* < 0.25 were further analyzed in a binary logistic regression model; ^*∗∗*^As specified by farmers; ^#^one-tailed Fisher's exact test; ^##^vaccination against hog cholera and/or haemorrhagic septicemia.

## Data Availability

The sets of data obtained and analyzed from this research are available from the corresponding author upon request.

## Supplementary Materials

Supplementary Materials Supplementary file: questionnaire for pig serum collection.
