# Supplementary material for: Individual and Herd-Level Seroprevalence in Association with Potential Risk Factors of Japanese Encephalitis in Pigs Collected from Urban, Periurban, and Rural Areas of Bali, Indonesia
Source: Vet Med Int. 2023 Feb 15;2023:9682657. doi: 10.1155/2023/9682657 (PMC9946736; doi:10.1155/2023/9682657)
Supplement: Supplementary Materials — Supplementary file: questionnaire for pig serum collection. [file 9682657.f1.docx]

# Supplementary file

| **Questionnaire for pig serum collection**  code of serum sample*:  (*protocol number is set based on the number pig sera, farm and its area collected). | |
| --- | --- |
| 1. **Individual identity** | |
| Address | Sub-district:  Districts: |
| 1. Age | - < 6 months - > 6 months |
| 1. Gender | - Male - Female |
| 1. Breed | - Local - Mixed - Other: |
| 1. Vaccination status of the animal (against any disease) | - Vaccinated,      - Non vaccinated |
| 1. Rearing management | |
| 1. Livestock rearing system | - traditional - semi-intensive |
| 2. Purpose of raising livestock | - investment - comsumption - cultural ceremony - other: .................. |
| 3. Total number of pigs per herd | ….. heads |
| 4.Pigs kept close to farmers’ house | - < 300 meters      - > 300 meters |
| 5. Pigpen size | - < 18 meter ^3^ - >18 meter ^3^ |
| 6. Number of pigs per pen | - < 6 - > 6 |
| 7. Is pigpen floor made of soil? | - Yes - No |
| 9. Frequency to clean the pigpen per day? | - Once - Twice - Three times - Other…. |
| 10. Is water sediment found on the pigpen floor? | - Yes - No |
| 11. Is there a pile of dung outside the pen? | - Yes - No |
| 12. Is there a drainage around the pigpen? | - Yes - No |
| 13. Cleanliness of the pigpen (on day visit) | - Clean - Dirty |
| 14. How is the dung/waste manage? | - Dumped outside pigherd - Thrown to ground |
| 15. Is the waste re-used? | - Yes (crops / biogas) - No |
| 16. Is faeces present in feed container? (on day visit) | - Yes - No |
| 17. Is faeces present in drinking container? | - Yes - No |
| 18. Are there any domesticated animals around the pigpens? | - Yes,   Please mention……   - No |
| 19. Is there paddy fileds around pigpen (< 500 meters)? | - Yes - No |
| 20. Is there one or more pigs being sick in the last 2 weeks? | - Yes, - No |
| 21. Is water from main pipe given to pigs? | - Yes - No |
| 22. Is there any natural water sources around the pigpen (< 500 meters)? | - Yes - No |
| 23. Is the feed and water provided in the same container? | - Separated - Mixed |
| 24. Is the feed/drink container clean (on day visit)? | - Clean - Dirty |
| 25. Frequency to clean the feed/drink container per day? | - Once - More than once |
| 26. Frequency of feeding per day? | - < twice - > twice |
| 27. Are kitchen leftover or food scraps fed to pig?? | - Yes - No |
| 28. Is the feed given cooked? | - Yes - No |
| 29. Have the pigs been sold in the last 3 month ? | - Yes - No |
| 30. Have pigs ever been sold outside the village? | - - Yes   - No |
| 31. Do pigs have potential direct contact with wild birds? | - Yes - No |
| 32. Are mosquitoes present around the pigpen? | - Yes - No |
